# *catena*-Poly[[[acetonitrile­copper(I)]-bis­[μ-bis­(diphenyl­phosphino)methane-κ^2^ *P*:*P*′]-copper(I)-μ-1,2-di-4-pyridy­l­ethene] bis­(tetra­fluoridoborate)]

**DOI:** 10.1107/S1600536809040896

**Published:** 2009-10-13

**Authors:** Yufei Wang, Xin Gan, Chen Jishu

**Affiliations:** a College of Chemistry and Chemical Engineering, Yunnan Normal University, Kunming 650092, People’s Republic of China; b Department of Chemistry and Life Science, Qujing Normal College, Qujing 655011, People’s Republic of China

## Abstract

The title dinuclear copper(I) complex, {[Cu_2_(C_2_H_3_N)(C_12_H_10_N_2_)(C_25_H_22_P_2_)_2_](BF_4_)_2_}_*n*_, contains 1,2-di-4-pyridyl­ethene, bis­(diphenyl­phosphino)methane and acetonitrile ligands. The two Cu atoms, one with an N_2_P_2_ ligand set and the other with an NP_2_ ligand set, are bridged by two bis­(diphenyl­phosphino)methane ligands, forming an eight-membered ring.

## Related literature

For the synthesis and structures of related compounds, see: Engelhardt *et al.* (1985 ▶); Fu *et al.* (2007 ▶).
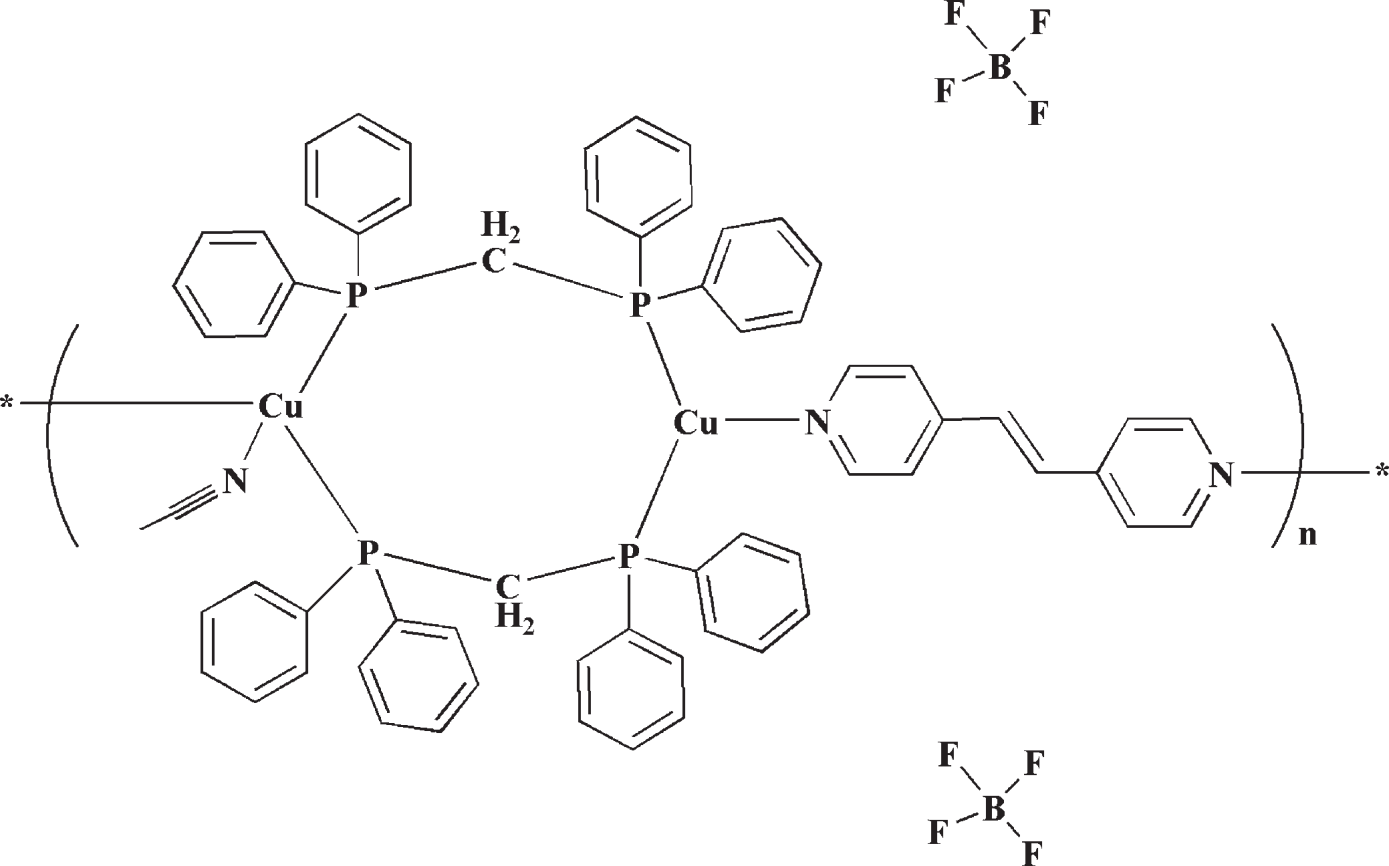


## Experimental

### 

#### Crystal data

[Cu_2_(C_2_H_3_N)(C_12_H_10_N_2_)(C_25_H_22_P_2_)_2_](BF_4_)_2_
*M* *_r_* = 1292.71
Triclinic, 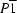

*a* = 11.146 (7) Å
*b* = 14.818 (9) Å
*c* = 18.532 (11) Å
α = 87.368 (10)°
β = 86.963 (11)°
γ = 84.992 (10)°
*V* = 3042 (3) Å^3^
*Z* = 2
Mo *K*α radiation
μ = 0.87 mm^−1^
*T* = 298 K
0.19 × 0.17 × 0.13 mm

#### Data collection

Bruker SMART diffractometer
Absorption correction: multi-scan (*SADABS*; Sheldrick, 1996 ▶) *T*_min_ = 0.852, *T*_max_ = 0.895
15646 measured reflections
10398 independent reflections
4625 reflections with *I* > 2σ(*I*)
*R*_int_ = 0.061

#### Refinement

*R*[*F* ^2^ > 2σ(*F* ^2^)] = 0.079
*wR*(*F* ^2^) = 0.152
*S* = 1.00
10398 reflections
749 parameters
39 restraints
H-atom parameters constrained
Δρ_max_ = 1.01 e Å^−3^
Δρ_min_ = −0.44 e Å^−3^

### 

Data collection: *SMART* (Siemens, 1996 ▶); cell refinement: *SAINT* (Siemens, 1996 ▶); data reduction: *SAINT*; program(s) used to solve structure: *SHELXS97* (Sheldrick, 2008 ▶); program(s) used to refine structure: *SHELXL97* (Sheldrick, 2008 ▶); molecular graphics: *ORTEP-3* (Farrugia, 1997 ▶) and *DIAMOND* (Brandenburg, 1998 ▶); software used to prepare material for publication: *SHELXTL* (Sheldrick, 2008 ▶).

## Supplementary Material

Crystal structure: contains datablocks I, global. DOI: 10.1107/S1600536809040896/jh2105sup1.cif

Structure factors: contains datablocks I. DOI: 10.1107/S1600536809040896/jh2105Isup2.hkl

Additional supplementary materials:  crystallographic information; 3D view; checkCIF report

## Figures and Tables

**Table:** 

Cu1—N3	2.050 (7)
Cu1—N1	2.135 (6)
Cu1—P2	2.279 (2)
Cu1—P1	2.283 (2)
Cu2—N2	2.060 (6)
Cu2—P3	2.239 (2)
Cu2—P4	2.251 (2)

**Table:** 

P2—Cu1—P1	125.82 (9)
P3—Cu2—P4	142.34 (8)

**Fig. 1.**
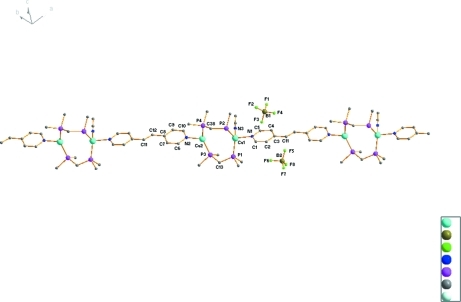
One-dimensional chain formed by Cu-N bonds (solid lines).

**Fig. 2.**
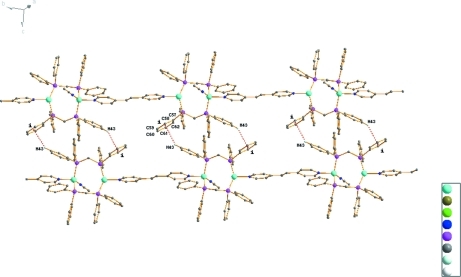
Two-dimensional network formed by connections between the chains through C—H···π interactions.

## supplementary crystallographic information

### Comment

The two copper atoms adopt different coordination geometries (Figure 1), one adopting a planar trigonal configuration and the other adopting a distroted tetrahedral geometry by the additional coordination of acetonitrile. The resulting NCu and N_2_Cu fragments are bridged by bis(diphenylphosphino)methane ligands and form a 1D chain (Fig. 1) furtherly linked together through C-H···π to form 2D network (Fig. 2). Similar compounds were obtained by Fu (Fu *et al.*, 2007, Engelhardt *et al.*, 1985). The average Cu-N and Cu-P distances are 2.082 Å and 2.263 Å, respectively, which are within the range of reference (Fu *et al.*, 2007). And the P-Cu-P angles of 125.82 (9) °, 142.34 (8) ° are larger than 115.85 (9)° (Engelhardt *et al.*, 1985).

### Experimental

Under nitrogen atmosphere, a mixture of bis(diphenylphosphino)methane (0.386 g, 1 mmol) and [Cu(CH_3_CN)_4_]BF_4_ (0.315 g, 1 mmol) in dichloromethane (30 mL) was stirred for 2 h at room temperature. Then 1,2-di-4-pyridylethene (0.091 g, 0.5 mmol) was added to the solution with stirring. After stirring the resulting solution for another 12 h, the solvents were removed and the residue was obtained. The pale yellow crystals were obtained by slow diffusion of diethyl ether into a dichloromethane solution of the complex.

### Refinement

All H atoms were positioned geometrically and refined as riding atoms, with C—H = 0.93,  Uiso(H) = 1.2Ueq(C).

### Crystal data

**Table:**

[Cu_2_(C_2_H_3_N)(C_12_H_10_N_2_)(C_25_H_22_P_2_)_2_](BF_4_)_2_	*Z* = 2
*M**_r_* = 1292.71	*F*(000) = 1324
Triclinic, *P*1	*D*_x_ = 1.411 Mg m^−^^3^
*a* = 11.146 (7) Å	Mo *K*α radiation, λ = 0.71073 Å
*b* = 14.818 (9) Å	Cell parameters from 2198 reflections
*c* = 18.532 (11) Å	θ = 3.2–21.0°
α = 87.368 (10)°	µ = 0.87 mm^−^^1^
β = 86.963 (11)°	*T* = 298 K
γ = 84.992 (10)°	Block, yellow
*V* = 3042 (3) Å^3^	0.19 × 0.17 × 0.13 mm

### Data collection

**Table:**

Bruker SMART diffractometer	10398 independent reflections
Radiation source: fine-focus sealed tube	4625 reflections with *I* > 2σ(*I*)
graphite	*R*_int_ = 0.061
φ and ω scans	θ_max_ = 25.0°, θ_min_ = 1.8°
Absorption correction: multi-scan (*SADABS*; Sheldrick, 1996)	*h* = −13→7
*T*_min_ = 0.852, *T*_max_ = 0.895	*k* = −17→17
15646 measured reflections	*l* = −22→21

### Refinement

**Table:**

Refinement on *F*^2^	Primary atom site location: structure-invariant direct methods
Least-squares matrix: full	Secondary atom site location: difference Fourier map
*R*[*F*^2^ > 2σ(*F*^2^)] = 0.079	Hydrogen site location: inferred from neighbouring sites
*wR*(*F*^2^) = 0.152	H-atom parameters constrained
*S* = 1.00	*w* = 1/[σ^2^(*F*_o_^2^) + (0.0373*P*)^2^] where *P* = (*F*_o_^2^ + 2*F*_c_^2^)/3
10398 reflections	(Δ/σ)_max_ = 0.001
749 parameters	Δρ_max_ = 1.00 e Å^−^^3^
39 restraints	Δρ_min_ = −0.44 e Å^−^^3^

### Special details

**Table:**

Geometry. All esds (except the esd in the dihedral angle between two l.s. planes) are estimated using the full covariance matrix. The cell esds are taken into account individually in the estimation of esds in distances, angles and torsion angles; correlations between esds in cell parameters are only used when they are defined by crystal symmetry. An approximate (isotropic) treatment of cell esds is used for estimating esds involving l.s. planes.
Refinement. Refinement of F^2^ against ALL reflections. The weighted R-factor wR and goodness of fit S are based on F^2^, conventional R-factors R are based on F, with F set to zero for negative F^2^. The threshold expression of F^2^ > 2sigma(F^2^) is used only for calculating R-factors(gt) etc. and is not relevant to the choice of reflections for refinement. R-factors based on F^2^ are statistically about twice as large as those based on F, and R- factors based on ALL data will be even larger.

### Fractional atomic coordinates and isotropic or equivalent isotropic displacement parameters (Å^2^)

**Table:**

	*x*	*y*	*z*	*U*_iso_*/*U*_eq_	
Cu1	0.27700 (8)	0.57866 (6)	0.24838 (5)	0.0411 (3)	
Cu2	0.04993 (8)	0.81487 (6)	0.24358 (5)	0.0474 (3)	
B1	0.7566 (10)	0.6129 (7)	0.2537 (6)	0.067 (3)	
B2	0.3676 (15)	0.0452 (9)	0.2281 (9)	0.139 (7)	
F1	0.8510 (5)	0.6714 (5)	0.2510 (3)	0.117 (2)	
F2	0.6789 (5)	0.6318 (4)	0.3101 (3)	0.0938 (17)	
F3	0.6988 (5)	0.6232 (4)	0.1921 (3)	0.1012 (19)	
F4	0.7995 (6)	0.5214 (5)	0.2598 (4)	0.166 (3)	
F5	0.4366 (8)	0.0476 (6)	0.2825 (5)	0.192 (4)	
F6	0.2843 (9)	0.1165 (7)	0.2408 (6)	0.224 (4)	
F7	0.3061 (6)	−0.0289 (4)	0.2262 (3)	0.120 (2)	
F8	0.4247 (10)	0.0703 (8)	0.1707 (5)	0.229 (4)	
N1	0.4004 (6)	0.4605 (4)	0.2560 (3)	0.0439 (16)	
N2	−0.0458 (6)	0.9402 (4)	0.2449 (3)	0.0474 (16)	
N3	0.3895 (6)	0.6814 (4)	0.2418 (3)	0.0436 (16)	
P1	0.18562 (17)	0.56636 (12)	0.14230 (10)	0.0389 (5)	
P2	0.18580 (17)	0.57121 (12)	0.36125 (10)	0.0402 (5)	
P3	0.06244 (17)	0.76465 (12)	0.13110 (11)	0.0424 (5)	
P4	0.05932 (17)	0.76720 (13)	0.36037 (11)	0.0437 (5)	
C1	0.3610 (8)	0.3770 (5)	0.2611 (4)	0.057 (2)	
H1	0.2779	0.3735	0.2639	0.068*	
C2	0.4310 (9)	0.2974 (5)	0.2625 (5)	0.068 (3)	
H2	0.3965	0.2423	0.2660	0.082*	
C3	0.5518 (10)	0.2997 (6)	0.2586 (4)	0.064 (3)	
C4	0.6005 (7)	0.3836 (6)	0.2543 (4)	0.053 (2)	
H4	0.6835	0.3872	0.2525	0.063*	
C5	0.5201 (7)	0.4630 (5)	0.2528 (4)	0.046 (2)	
H5	0.5518	0.5191	0.2494	0.055*	
C6	−0.1647 (8)	0.9443 (5)	0.2425 (4)	0.058 (2)	
H6	−0.2005	0.8907	0.2374	0.070*	
C7	−0.2378 (7)	1.0234 (6)	0.2471 (4)	0.065 (2)	
H7	−0.3211	1.0226	0.2462	0.078*	
C8	−0.1878 (9)	1.1032 (6)	0.2530 (4)	0.059 (2)	
C9	−0.0659 (8)	1.1002 (5)	0.2536 (4)	0.066 (3)	
H9	−0.0282	1.1533	0.2571	0.079*	
C10	0.0007 (8)	1.0197 (5)	0.2491 (4)	0.062 (2)	
H10	0.0841	1.0197	0.2490	0.074*	
C11	−0.3744 (9)	1.2097 (6)	0.2578 (5)	0.076 (3)	
H11	−0.4172	1.1584	0.2591	0.092*	
C12	−0.2620 (10)	1.1961 (7)	0.2555 (5)	0.079 (3)	
H12	−0.2188	1.2471	0.2553	0.095*	
C13	0.0543 (6)	0.6422 (4)	0.1200 (4)	0.0437 (19)	
H13A	−0.0141	0.6232	0.1496	0.052*	
H13B	0.0376	0.6337	0.0700	0.052*	
C14	0.1318 (7)	0.4541 (5)	0.1332 (4)	0.0420 (19)	
C15	0.0127 (7)	0.4378 (5)	0.1429 (4)	0.057 (2)	
H15	−0.0452	0.4856	0.1516	0.068*	
C16	−0.0220 (9)	0.3501 (7)	0.1397 (5)	0.086 (3)	
H16	−0.1027	0.3396	0.1480	0.103*	
C17	0.0615 (11)	0.2789 (6)	0.1244 (5)	0.079 (3)	
H17	0.0387	0.2203	0.1213	0.095*	
C18	0.1802 (10)	0.2974 (6)	0.1140 (4)	0.073 (3)	
H18	0.2385	0.2502	0.1041	0.088*	
C19	0.2142 (8)	0.3822 (5)	0.1176 (4)	0.067 (3)	
H19	0.2951	0.3923	0.1093	0.081*	
C20	0.2829 (6)	0.5734 (4)	0.0609 (4)	0.0394 (18)	
C21	0.3966 (7)	0.6086 (5)	0.0635 (4)	0.051 (2)	
H21	0.4234	0.6236	0.1077	0.061*	
C22	0.4675 (7)	0.6207 (5)	0.0020 (4)	0.056 (2)	
H22	0.5389	0.6482	0.0046	0.068*	
C23	0.4357 (8)	0.5934 (5)	−0.0630 (4)	0.061 (2)	
H23	0.4862	0.5993	−0.1043	0.073*	
C24	0.3240 (8)	0.5561 (6)	−0.0659 (4)	0.065 (2)	
H24	0.2997	0.5374	−0.1096	0.078*	
C25	0.2520 (7)	0.5473 (5)	−0.0053 (4)	0.053 (2)	
H25	0.1787	0.5226	−0.0086	0.064*	
C26	0.1855 (6)	0.7985 (4)	0.0709 (4)	0.0395 (18)	
C27	0.1913 (7)	0.7824 (5)	−0.0034 (4)	0.051 (2)	
H27	0.1323	0.7514	−0.0228	0.061*	
C28	0.2841 (9)	0.8123 (6)	−0.0474 (5)	0.065 (2)	
H28	0.2865	0.8026	−0.0967	0.077*	
C29	0.3739 (8)	0.8567 (6)	−0.0191 (6)	0.076 (3)	
H29	0.4374	0.8756	−0.0489	0.091*	
C30	0.3685 (7)	0.8729 (5)	0.0539 (6)	0.062 (2)	
H30	0.4283	0.9031	0.0733	0.075*	
C31	0.2757 (7)	0.8447 (5)	0.0974 (4)	0.053 (2)	
H31	0.2726	0.8568	0.1463	0.063*	
C32	−0.0722 (7)	0.8126 (5)	0.0853 (4)	0.051 (2)	
C33	−0.1728 (8)	0.7703 (5)	0.0819 (4)	0.058 (2)	
H33	−0.1736	0.7107	0.1001	0.069*	
C34	−0.2757 (8)	0.8122 (6)	0.0522 (5)	0.071 (3)	
H34	−0.3460	0.7824	0.0540	0.085*	
C35	−0.2744 (8)	0.8942 (7)	0.0215 (5)	0.089 (3)	
H35	−0.3410	0.9196	−0.0028	0.107*	
C36	−0.1746 (9)	0.9421 (6)	0.0252 (5)	0.086 (3)	
H36	−0.1762	1.0021	0.0079	0.103*	
C37	−0.0730 (8)	0.9002 (6)	0.0547 (5)	0.079 (3)	
H37	−0.0036	0.9308	0.0542	0.095*	
C38	0.0500 (6)	0.6457 (4)	0.3867 (4)	0.049 (2)	
H38A	0.0353	0.6400	0.4388	0.058*	
H38B	−0.0187	0.6245	0.3645	0.058*	
C39	0.1300 (7)	0.4593 (5)	0.3766 (4)	0.049 (2)	
C40	0.0297 (8)	0.4385 (6)	0.3444 (4)	0.064 (2)	
H40	−0.0177	0.4843	0.3208	0.077*	
C41	−0.0033 (10)	0.3502 (8)	0.3460 (6)	0.102 (4)	
H41	−0.0726	0.3381	0.3236	0.122*	
C42	0.0629 (12)	0.2815 (7)	0.3793 (5)	0.092 (4)	
H42	0.0416	0.2222	0.3788	0.110*	
C43	0.1606 (11)	0.3010 (6)	0.4133 (5)	0.092 (3)	
H43	0.2065	0.2547	0.4372	0.111*	
C44	0.1940 (9)	0.3887 (6)	0.4133 (5)	0.078 (3)	
H44	0.2606	0.4005	0.4383	0.093*	
C45	0.2827 (7)	0.5788 (4)	0.4352 (4)	0.0406 (19)	
C46	0.2484 (8)	0.5600 (5)	0.5079 (4)	0.060 (2)	
H46	0.1726	0.5398	0.5188	0.071*	
C47	0.3213 (9)	0.5700 (6)	0.5632 (4)	0.063 (2)	
H47	0.2954	0.5552	0.6105	0.075*	
C48	0.4328 (9)	0.6016 (5)	0.5495 (5)	0.062 (2)	
H48	0.4818	0.6106	0.5872	0.074*	
C49	0.4699 (8)	0.6195 (5)	0.4797 (5)	0.062 (2)	
H49	0.5456	0.6402	0.4695	0.075*	
C50	0.3962 (8)	0.6074 (5)	0.4233 (4)	0.051 (2)	
H50	0.4247	0.6190	0.3760	0.062*	
C51	0.1817 (7)	0.8027 (5)	0.4107 (4)	0.048 (2)	
C52	0.1880 (8)	0.7839 (5)	0.4865 (4)	0.057 (2)	
H52	0.1293	0.7523	0.5116	0.069*	
C53	0.2820 (9)	0.8128 (6)	0.5225 (5)	0.068 (3)	
H53	0.2881	0.7987	0.5717	0.082*	
C54	0.3647 (9)	0.8613 (6)	0.4871 (7)	0.074 (3)	
H54	0.4271	0.8808	0.5121	0.089*	
C55	0.3583 (8)	0.8822 (5)	0.4150 (6)	0.068 (3)	
H55	0.4167	0.9154	0.3911	0.082*	
C56	0.2644 (7)	0.8540 (5)	0.3768 (5)	0.057 (2)	
H56	0.2585	0.8705	0.3280	0.068*	
C57	−0.0764 (7)	0.8199 (5)	0.4061 (4)	0.049 (2)	
C58	−0.0741 (8)	0.8976 (6)	0.4431 (5)	0.084 (3)	
H58	−0.0010	0.9216	0.4488	0.101*	
C59	−0.1805 (9)	0.9410 (7)	0.4721 (6)	0.096 (3)	
H59	−0.1778	0.9944	0.4963	0.115*	
C60	−0.2890 (9)	0.9062 (7)	0.4654 (6)	0.099 (3)	
H60	−0.3598	0.9354	0.4849	0.119*	
C61	−0.2917 (8)	0.8273 (6)	0.4294 (5)	0.082 (3)	
H61	−0.3650	0.8031	0.4247	0.098*	
C62	−0.1892 (8)	0.7843 (6)	0.4007 (4)	0.068 (2)	
H62	−0.1930	0.7306	0.3770	0.081*	
C63	0.4518 (7)	0.7361 (5)	0.2385 (4)	0.045 (2)	
C64	0.5343 (8)	0.8084 (6)	0.2355 (5)	0.082 (3)	
H64A	0.5945	0.7979	0.1971	0.123*	
H64B	0.5727	0.8086	0.2806	0.123*	
H64C	0.4897	0.8659	0.2267	0.123*	

### Atomic displacement parameters (Å^2^)

**Table:**

	*U*^11^	*U*^22^	*U*^33^	*U*^12^	*U*^13^	*U*^23^
Cu1	0.0400 (6)	0.0310 (5)	0.0509 (6)	0.0062 (4)	−0.0044 (5)	−0.0040 (4)
Cu2	0.0430 (6)	0.0369 (6)	0.0598 (7)	0.0136 (5)	−0.0053 (5)	−0.0046 (4)
B1	0.069 (8)	0.065 (8)	0.065 (8)	−0.005 (7)	0.002 (7)	−0.003 (6)
B2	0.148 (18)	0.038 (9)	0.23 (2)	−0.026 (10)	0.023 (17)	−0.003 (11)
F1	0.095 (3)	0.162 (4)	0.101 (3)	−0.046 (3)	0.000 (3)	−0.020 (3)
F2	0.085 (4)	0.101 (4)	0.098 (4)	−0.025 (3)	0.024 (3)	−0.030 (3)
F3	0.112 (5)	0.108 (5)	0.092 (4)	−0.045 (4)	−0.021 (4)	0.001 (3)
F4	0.094 (5)	0.143 (7)	0.247 (8)	0.063 (5)	−0.006 (5)	0.016 (6)
F5	0.183 (9)	0.185 (9)	0.227 (9)	−0.105 (7)	−0.089 (7)	0.032 (7)
F6	0.207 (8)	0.130 (6)	0.338 (9)	−0.019 (6)	−0.028 (7)	−0.032 (6)
F7	0.130 (5)	0.063 (4)	0.169 (6)	−0.018 (4)	−0.015 (4)	−0.015 (4)
F8	0.224 (8)	0.289 (9)	0.189 (7)	−0.141 (7)	0.021 (6)	0.004 (6)
N1	0.040 (4)	0.037 (4)	0.054 (4)	0.012 (3)	−0.006 (3)	−0.012 (3)
N2	0.044 (4)	0.028 (4)	0.068 (5)	0.010 (3)	−0.006 (4)	−0.002 (3)
N3	0.048 (4)	0.042 (4)	0.040 (4)	0.012 (3)	−0.012 (3)	−0.009 (3)
P1	0.0406 (12)	0.0274 (11)	0.0478 (12)	0.0075 (9)	−0.0082 (10)	−0.0054 (9)
P2	0.0415 (12)	0.0320 (11)	0.0454 (12)	0.0094 (9)	−0.0062 (10)	−0.0040 (9)
P3	0.0364 (12)	0.0313 (11)	0.0576 (14)	0.0068 (9)	−0.0045 (10)	0.0021 (9)
P4	0.0377 (12)	0.0374 (12)	0.0543 (13)	0.0088 (10)	−0.0010 (10)	−0.0081 (10)
C1	0.063 (6)	0.036 (5)	0.071 (6)	0.001 (5)	−0.004 (5)	−0.007 (4)
C2	0.066 (7)	0.029 (5)	0.108 (8)	0.013 (5)	−0.004 (6)	−0.010 (5)
C3	0.088 (8)	0.062 (7)	0.035 (5)	0.034 (6)	−0.003 (5)	−0.007 (4)
C4	0.034 (5)	0.077 (7)	0.044 (5)	0.018 (5)	−0.005 (4)	−0.012 (4)
C5	0.052 (6)	0.040 (5)	0.045 (5)	0.006 (4)	0.001 (4)	−0.005 (4)
C6	0.055 (6)	0.026 (5)	0.095 (7)	−0.004 (4)	−0.009 (5)	−0.012 (4)
C7	0.035 (5)	0.075 (7)	0.079 (7)	0.023 (5)	0.001 (5)	0.001 (5)
C8	0.078 (7)	0.042 (5)	0.054 (6)	0.017 (5)	−0.008 (5)	−0.005 (4)
C9	0.062 (7)	0.034 (5)	0.100 (7)	0.011 (5)	−0.016 (6)	−0.004 (5)
C10	0.071 (6)	0.036 (5)	0.077 (6)	0.008 (5)	−0.010 (5)	−0.002 (4)
C11	0.083 (8)	0.056 (6)	0.086 (7)	0.012 (6)	−0.001 (6)	0.003 (5)
C12	0.082 (8)	0.078 (7)	0.071 (7)	0.029 (6)	−0.010 (6)	−0.008 (5)
C13	0.034 (4)	0.045 (5)	0.051 (5)	0.004 (4)	−0.007 (4)	0.004 (4)
C14	0.047 (5)	0.033 (4)	0.046 (5)	−0.005 (4)	−0.008 (4)	0.004 (3)
C15	0.050 (6)	0.051 (6)	0.071 (6)	−0.004 (5)	−0.009 (5)	−0.001 (4)
C16	0.074 (7)	0.061 (7)	0.124 (9)	−0.029 (6)	0.007 (6)	0.006 (6)
C17	0.109 (9)	0.039 (6)	0.095 (8)	−0.018 (6)	−0.027 (7)	0.004 (5)
C18	0.103 (9)	0.045 (6)	0.072 (7)	0.010 (6)	−0.014 (6)	−0.020 (5)
C19	0.067 (6)	0.033 (5)	0.101 (7)	0.007 (5)	0.001 (5)	−0.018 (5)
C20	0.029 (5)	0.028 (4)	0.060 (5)	0.012 (3)	−0.006 (4)	−0.011 (4)
C21	0.060 (6)	0.052 (5)	0.039 (5)	0.005 (4)	0.002 (4)	−0.003 (4)
C22	0.055 (6)	0.051 (5)	0.063 (6)	−0.006 (4)	0.004 (5)	−0.009 (4)
C23	0.060 (6)	0.072 (6)	0.047 (6)	0.004 (5)	0.012 (5)	−0.013 (4)
C24	0.059 (6)	0.085 (7)	0.049 (6)	0.005 (5)	−0.005 (5)	−0.013 (5)
C25	0.058 (6)	0.053 (5)	0.049 (5)	−0.001 (4)	0.001 (5)	−0.011 (4)
C26	0.034 (5)	0.025 (4)	0.058 (5)	0.006 (3)	−0.007 (4)	0.002 (4)
C27	0.054 (6)	0.038 (5)	0.059 (6)	0.005 (4)	−0.012 (5)	−0.001 (4)
C28	0.065 (7)	0.061 (6)	0.062 (6)	0.011 (5)	0.009 (6)	0.012 (5)
C29	0.040 (6)	0.076 (7)	0.106 (9)	0.003 (5)	0.018 (6)	0.030 (6)
C30	0.032 (5)	0.051 (6)	0.103 (8)	−0.004 (4)	−0.007 (5)	0.012 (5)
C31	0.038 (5)	0.046 (5)	0.072 (6)	0.005 (4)	−0.016 (5)	0.009 (4)
C32	0.046 (5)	0.030 (5)	0.075 (6)	0.008 (4)	−0.002 (4)	0.004 (4)
C33	0.053 (6)	0.047 (5)	0.071 (6)	0.005 (5)	−0.017 (5)	0.012 (4)
C34	0.046 (6)	0.057 (6)	0.110 (8)	−0.005 (5)	−0.015 (5)	0.012 (5)
C35	0.048 (7)	0.106 (9)	0.107 (8)	0.016 (6)	−0.028 (6)	0.035 (7)
C36	0.065 (6)	0.062 (5)	0.126 (7)	0.006 (5)	−0.017 (5)	0.044 (5)
C37	0.045 (6)	0.050 (6)	0.139 (9)	0.002 (5)	−0.014 (6)	0.021 (6)
C38	0.038 (5)	0.042 (5)	0.065 (5)	0.010 (4)	−0.010 (4)	−0.011 (4)
C39	0.056 (6)	0.040 (5)	0.049 (5)	0.003 (4)	0.004 (4)	0.001 (4)
C40	0.059 (6)	0.046 (6)	0.087 (7)	0.004 (5)	−0.020 (5)	0.006 (5)
C41	0.093 (9)	0.066 (8)	0.154 (11)	−0.029 (7)	−0.033 (8)	−0.012 (7)
C42	0.136 (11)	0.055 (7)	0.089 (8)	−0.038 (8)	0.000 (8)	−0.012 (6)
C43	0.142 (11)	0.034 (6)	0.101 (8)	−0.001 (6)	−0.018 (8)	−0.003 (5)
C44	0.092 (8)	0.054 (6)	0.088 (7)	0.000 (6)	−0.034 (6)	0.009 (5)
C45	0.032 (5)	0.023 (4)	0.066 (6)	0.012 (3)	−0.012 (4)	−0.005 (4)
C46	0.069 (6)	0.053 (5)	0.055 (6)	0.004 (5)	0.000 (5)	−0.006 (4)
C47	0.078 (7)	0.066 (6)	0.043 (6)	0.004 (5)	−0.006 (5)	−0.013 (4)
C48	0.073 (7)	0.058 (6)	0.056 (6)	0.005 (5)	−0.028 (5)	−0.010 (5)
C49	0.064 (6)	0.048 (5)	0.074 (7)	0.006 (4)	−0.013 (6)	−0.002 (5)
C50	0.056 (6)	0.050 (5)	0.045 (5)	0.014 (4)	−0.014 (5)	0.000 (4)
C51	0.037 (5)	0.041 (5)	0.062 (6)	0.009 (4)	0.008 (4)	−0.010 (4)
C52	0.059 (6)	0.045 (5)	0.065 (6)	0.012 (4)	−0.006 (5)	−0.014 (4)
C53	0.076 (7)	0.057 (6)	0.072 (7)	0.026 (5)	−0.036 (6)	−0.021 (5)
C54	0.045 (6)	0.053 (6)	0.127 (10)	0.008 (5)	−0.023 (7)	−0.023 (6)
C55	0.044 (6)	0.052 (6)	0.107 (8)	−0.004 (5)	0.009 (6)	−0.012 (6)
C56	0.044 (5)	0.042 (5)	0.085 (6)	−0.009 (4)	0.013 (5)	−0.013 (4)
C57	0.049 (5)	0.032 (5)	0.064 (6)	0.018 (4)	−0.004 (4)	−0.010 (4)
C58	0.045 (6)	0.051 (6)	0.157 (10)	0.022 (5)	−0.006 (6)	−0.041 (6)
C59	0.073 (6)	0.073 (6)	0.143 (7)	−0.005 (5)	0.011 (6)	−0.042 (5)
C60	0.059 (7)	0.082 (8)	0.158 (10)	0.011 (6)	0.012 (7)	−0.060 (7)
C61	0.037 (6)	0.077 (7)	0.130 (9)	0.009 (5)	0.004 (6)	−0.031 (6)
C62	0.048 (6)	0.071 (6)	0.084 (7)	0.004 (5)	−0.005 (5)	−0.030 (5)
C63	0.039 (5)	0.037 (5)	0.058 (5)	−0.001 (4)	−0.006 (4)	−0.002 (4)
C64	0.070 (7)	0.056 (6)	0.124 (8)	−0.013 (5)	−0.006 (6)	−0.014 (5)

### Geometric parameters (Å, °)

**Table:**

Cu1—N3	2.050 (7)	C24—C25	1.353 (10)
Cu1—N1	2.135 (6)	C24—H24	0.9300
Cu1—P2	2.279 (2)	C25—H25	0.9300
Cu1—P1	2.283 (2)	C26—C31	1.386 (9)
Cu2—N2	2.060 (6)	C26—C27	1.405 (9)
Cu2—P3	2.239 (2)	C27—C28	1.375 (10)
Cu2—P4	2.251 (2)	C27—H27	0.9300
B1—F3	1.338 (10)	C28—C29	1.383 (11)
B1—F2	1.344 (10)	C28—H28	0.9300
B1—F4	1.400 (10)	C29—C30	1.381 (11)
B1—F1	1.418 (10)	C29—H29	0.9300
B2—F8	1.268 (13)	C30—C31	1.360 (10)
B2—F5	1.304 (15)	C30—H30	0.9300
B2—F7	1.347 (12)	C31—H31	0.9300
B2—F6	1.364 (13)	C32—C33	1.338 (9)
N1—C5	1.335 (8)	C32—C37	1.392 (10)
N1—C1	1.347 (8)	C33—C34	1.384 (10)
N2—C6	1.325 (9)	C33—H33	0.9300
N2—C10	1.335 (9)	C34—C35	1.319 (11)
N3—C63	1.110 (8)	C34—H34	0.9300
P1—C20	1.815 (7)	C35—C36	1.377 (11)
P1—C13	1.820 (7)	C35—H35	0.9300
P1—C14	1.834 (7)	C36—C37	1.371 (10)
P2—C45	1.803 (7)	C36—H36	0.9300
P2—C39	1.827 (8)	C37—H37	0.9300
P2—C38	1.847 (7)	C38—H38A	0.9700
P3—C26	1.812 (7)	C38—H38B	0.9700
P3—C32	1.836 (8)	C39—C40	1.360 (10)
P3—C13	1.846 (7)	C39—C44	1.386 (10)
P4—C51	1.820 (8)	C40—C41	1.388 (11)
P4—C57	1.826 (7)	C40—H40	0.9300
P4—C38	1.855 (7)	C41—C42	1.350 (12)
C1—C2	1.357 (10)	C41—H41	0.9300
C1—H1	0.9300	C42—C43	1.346 (12)
C2—C3	1.348 (11)	C42—H42	0.9300
C2—H2	0.9300	C43—C44	1.382 (11)
C3—C4	1.396 (11)	C43—H43	0.9300
C3—C11^i^	1.506 (11)	C44—H44	0.9300
C4—C5	1.415 (10)	C45—C50	1.374 (10)
C4—H4	0.9300	C45—C46	1.402 (9)
C5—H5	0.9300	C46—C47	1.362 (10)
C6—C7	1.372 (10)	C46—H46	0.9300
C6—H6	0.9300	C47—C48	1.373 (11)
C7—C8	1.361 (10)	C47—H47	0.9300
C7—H7	0.9300	C48—C49	1.360 (10)
C8—C9	1.356 (11)	C48—H48	0.9300
C8—C12	1.545 (11)	C49—C50	1.390 (9)
C9—C10	1.352 (10)	C49—H49	0.9300
C9—H9	0.9300	C50—H50	0.9300
C10—H10	0.9300	C51—C56	1.356 (9)
C11—C12	1.250 (10)	C51—C52	1.424 (9)
C11—C3^ii^	1.506 (11)	C52—C53	1.380 (10)
C11—H11	0.9300	C52—H52	0.9300
C12—H12	0.9300	C53—C54	1.344 (11)
C13—H13A	0.9700	C53—H53	0.9300
C13—H13B	0.9700	C54—C55	1.362 (11)
C14—C15	1.371 (10)	C54—H54	0.9300
C14—C19	1.375 (10)	C55—C56	1.398 (10)
C15—C16	1.393 (11)	C55—H55	0.9300
C15—H15	0.9300	C56—H56	0.9300
C16—C17	1.373 (12)	C57—C58	1.370 (10)
C16—H16	0.9300	C57—C62	1.416 (10)
C17—C18	1.377 (12)	C58—C59	1.393 (11)
C17—H17	0.9300	C58—H58	0.9300
C18—C19	1.349 (10)	C59—C60	1.370 (11)
C18—H18	0.9300	C59—H59	0.9300
C19—H19	0.9300	C60—C61	1.377 (11)
C20—C25	1.373 (9)	C60—H60	0.9300
C20—C21	1.415 (9)	C61—C62	1.354 (11)
C21—C22	1.367 (9)	C61—H61	0.9300
C21—H21	0.9300	C62—H62	0.9300
C22—C23	1.362 (9)	C63—C64	1.468 (10)
C22—H22	0.9300	C64—H64A	0.9600
C23—C24	1.411 (10)	C64—H64B	0.9600
C23—H23	0.9300	C64—H64C	0.9600
			
N3—Cu1—N1	102.5 (2)	C23—C24—H24	119.8
N3—Cu1—P2	109.40 (16)	C24—C25—C20	122.5 (8)
N1—Cu1—P2	99.37 (17)	C24—C25—H25	118.7
N3—Cu1—P1	111.69 (16)	C20—C25—H25	118.7
N1—Cu1—P1	104.49 (16)	C31—C26—C27	117.9 (7)
P2—Cu1—P1	125.82 (9)	C31—C26—P3	119.9 (6)
N2—Cu2—P3	109.65 (17)	C27—C26—P3	122.1 (6)
N2—Cu2—P4	105.34 (17)	C28—C27—C26	120.0 (8)
P3—Cu2—P4	142.34 (8)	C28—C27—H27	120.0
F3—B1—F2	109.8 (9)	C26—C27—H27	120.0
F3—B1—F4	106.2 (8)	C27—C28—C29	120.6 (8)
F2—B1—F4	108.7 (8)	C27—C28—H28	119.7
F3—B1—F1	109.4 (8)	C29—C28—H28	119.7
F2—B1—F1	110.3 (8)	C30—C29—C28	119.5 (8)
F4—B1—F1	112.3 (9)	C30—C29—H29	120.2
F8—B2—F5	108.9 (13)	C28—C29—H29	120.2
F8—B2—F7	117.9 (14)	C31—C30—C29	120.0 (8)
F5—B2—F7	115.8 (12)	C31—C30—H30	120.0
F8—B2—F6	103.6 (12)	C29—C30—H30	120.0
F5—B2—F6	101.8 (13)	C30—C31—C26	121.9 (8)
F7—B2—F6	106.6 (12)	C30—C31—H31	119.0
C5—N1—C1	115.4 (7)	C26—C31—H31	119.0
C5—N1—Cu1	123.5 (5)	C33—C32—C37	117.1 (7)
C1—N1—Cu1	121.0 (5)	C33—C32—P3	124.2 (6)
C6—N2—C10	115.5 (7)	C37—C32—P3	118.6 (7)
C6—N2—Cu2	118.5 (5)	C32—C33—C34	122.1 (8)
C10—N2—Cu2	126.0 (6)	C32—C33—H33	119.0
C63—N3—Cu1	178.9 (6)	C34—C33—H33	119.0
C20—P1—C13	102.9 (3)	C35—C34—C33	120.2 (8)
C20—P1—C14	100.0 (3)	C35—C34—H34	119.9
C13—P1—C14	102.4 (3)	C33—C34—H34	119.9
C20—P1—Cu1	115.6 (2)	C34—C35—C36	120.1 (8)
C13—P1—Cu1	120.4 (2)	C34—C35—H35	120.0
C14—P1—Cu1	112.9 (2)	C36—C35—H35	120.0
C45—P2—C39	103.4 (3)	C37—C36—C35	119.1 (8)
C45—P2—C38	103.6 (3)	C37—C36—H36	120.4
C39—P2—C38	101.2 (3)	C35—C36—H36	120.4
C45—P2—Cu1	115.8 (3)	C36—C37—C32	121.0 (8)
C39—P2—Cu1	108.5 (2)	C36—C37—H37	119.5
C38—P2—Cu1	122.0 (2)	C32—C37—H37	119.5
C26—P3—C32	103.3 (3)	P2—C38—P4	114.6 (4)
C26—P3—C13	106.5 (3)	P2—C38—H38A	108.6
C32—P3—C13	101.3 (3)	P4—C38—H38A	108.6
C26—P3—Cu2	117.8 (3)	P2—C38—H38B	108.6
C32—P3—Cu2	107.8 (3)	P4—C38—H38B	108.6
C13—P3—Cu2	118.0 (2)	H38A—C38—H38B	107.6
C51—P4—C57	103.9 (3)	C40—C39—C44	116.5 (8)
C51—P4—C38	105.3 (3)	C40—C39—P2	120.4 (6)
C57—P4—C38	101.9 (3)	C44—C39—P2	122.6 (7)
C51—P4—Cu2	117.5 (3)	C39—C40—C41	121.3 (8)
C57—P4—Cu2	105.6 (2)	C39—C40—H40	119.4
C38—P4—Cu2	120.3 (2)	C41—C40—H40	119.4
N1—C1—C2	126.1 (8)	C42—C41—C40	121.5 (10)
N1—C1—H1	117.0	C42—C41—H41	119.3
C2—C1—H1	117.0	C40—C41—H41	119.3
C3—C2—C1	118.6 (8)	C43—C42—C41	118.2 (10)
C3—C2—H2	120.7	C43—C42—H42	120.9
C1—C2—H2	120.7	C41—C42—H42	120.9
C2—C3—C4	119.1 (8)	C42—C43—C44	121.2 (10)
C2—C3—C11^i^	116.6 (9)	C42—C43—H43	119.4
C4—C3—C11^i^	124.3 (9)	C44—C43—H43	119.4
C3—C4—C5	118.2 (8)	C43—C44—C39	121.3 (9)
C3—C4—H4	120.9	C43—C44—H44	119.4
C5—C4—H4	120.9	C39—C44—H44	119.4
N1—C5—C4	122.6 (7)	C50—C45—C46	115.2 (7)
N1—C5—H5	118.7	C50—C45—P2	120.8 (6)
C4—C5—H5	118.7	C46—C45—P2	123.9 (6)
N2—C6—C7	123.5 (7)	C47—C46—C45	122.9 (8)
N2—C6—H6	118.3	C47—C46—H46	118.5
C7—C6—H6	118.3	C45—C46—H46	118.5
C8—C7—C6	119.6 (8)	C46—C47—C48	120.4 (8)
C8—C7—H7	120.2	C46—C47—H47	119.8
C6—C7—H7	120.2	C48—C47—H47	119.8
C9—C8—C7	117.5 (8)	C49—C48—C47	118.5 (8)
C9—C8—C12	119.0 (9)	C49—C48—H48	120.8
C7—C8—C12	123.4 (9)	C47—C48—H48	120.8
C10—C9—C8	119.8 (8)	C48—C49—C50	120.9 (8)
C10—C9—H9	120.1	C48—C49—H49	119.5
C8—C9—H9	120.1	C50—C49—H49	119.5
N2—C10—C9	124.2 (8)	C45—C50—C49	122.1 (8)
N2—C10—H10	117.9	C45—C50—H50	118.9
C9—C10—H10	117.9	C49—C50—H50	118.9
C12—C11—C3^ii^	127.2 (10)	C56—C51—C52	118.6 (7)
C12—C11—H11	116.4	C56—C51—P4	119.7 (6)
C3^ii^—C11—H11	116.4	C52—C51—P4	121.6 (6)
C11—C12—C8	126.5 (10)	C53—C52—C51	119.5 (8)
C11—C12—H12	116.8	C53—C52—H52	120.2
C8—C12—H12	116.8	C51—C52—H52	120.2
P1—C13—P3	117.6 (4)	C54—C53—C52	120.5 (9)
P1—C13—H13A	107.9	C54—C53—H53	119.7
P3—C13—H13A	107.9	C52—C53—H53	119.7
P1—C13—H13B	107.9	C53—C54—C55	120.9 (9)
P3—C13—H13B	107.9	C53—C54—H54	119.6
H13A—C13—H13B	107.2	C55—C54—H54	119.6
C15—C14—C19	118.0 (7)	C54—C55—C56	120.1 (8)
C15—C14—P1	123.0 (6)	C54—C55—H55	119.9
C19—C14—P1	119.0 (6)	C56—C55—H55	119.9
C14—C15—C16	120.5 (8)	C51—C56—C55	120.3 (8)
C14—C15—H15	119.8	C51—C56—H56	119.9
C16—C15—H15	119.8	C55—C56—H56	119.9
C17—C16—C15	120.8 (9)	C58—C57—C62	117.8 (7)
C17—C16—H16	119.6	C58—C57—P4	121.5 (7)
C15—C16—H16	119.6	C62—C57—P4	120.6 (6)
C16—C17—C18	117.5 (9)	C57—C58—C59	120.4 (8)
C16—C17—H17	121.3	C57—C58—H58	119.8
C18—C17—H17	121.3	C59—C58—H58	119.8
C19—C18—C17	121.8 (9)	C60—C59—C58	120.9 (9)
C19—C18—H18	119.1	C60—C59—H59	119.6
C17—C18—H18	119.1	C58—C59—H59	119.6
C18—C19—C14	121.4 (9)	C59—C60—C61	119.0 (9)
C18—C19—H19	119.3	C59—C60—H60	120.5
C14—C19—H19	119.3	C61—C60—H60	120.5
C25—C20—C21	116.6 (7)	C62—C61—C60	121.0 (9)
C25—C20—P1	123.5 (6)	C62—C61—H61	119.5
C21—C20—P1	119.9 (5)	C60—C61—H61	119.5
C22—C21—C20	121.0 (7)	C61—C62—C57	120.9 (8)
C22—C21—H21	119.5	C61—C62—H62	119.6
C20—C21—H21	119.5	C57—C62—H62	119.6
C23—C22—C21	121.4 (8)	N3—C63—C64	179.1 (8)
C23—C22—H22	119.3	C63—C64—H64A	109.5
C21—C22—H22	119.3	C63—C64—H64B	109.5
C22—C23—C24	117.9 (8)	H64A—C64—H64B	109.5
C22—C23—H23	121.0	C63—C64—H64C	109.5
C24—C23—H23	121.0	H64A—C64—H64C	109.5
C25—C24—C23	120.4 (8)	H64B—C64—H64C	109.5
C25—C24—H24	119.8		

Symmetry codes: (i) *x*+1, *y*−1, *z*; (ii) *x*−1, *y*+1, *z*.
